# Trajectories of Cognitive Decline and Subsequent Physical Activity

**DOI:** 10.1093/geroni/igaf122.1951

**Published:** 2025-12-31

**Authors:** Jennifer Schrack, Sunan Gao, Ciprian Crainiceanu, Anis Davoudi, Ryan Dougherty, Lacey Etzkorn, Amal Wanigatunga, Adam Spira

**Affiliations:** Johns Hopkins Bloomberg School of Public Health, Baltimore, Maryland, United States; Johns Hopkins University, Baltimore, Maryland, United States; Johns Hopkins University, Baltimore, Maryland, United States; Johns Hopkins University, Baltimore, Maryland, United States; Rutgers University, New Brunswick, New Jersey, United States; Johns Hopkins Bloomberg School of Public Health, Baltimore, Maryland, United States; Johns Hopkins Bloomberg School of Public Health, Baltimore, Maryland, United States

## Abstract

Physical activity (PA) is a modifiable risk factor for dementia, but whether a faster rate of cognitive decline adversely affects subsequent PA is unknown. We investigated the association of cognitive trajectories over 8.2((SD = 1.6), visits 5-9) years with late-life PA (visit 9) in 810 (mean age73.1(SD = 4.0)years, 60.4%, female) Atherosclerosis Risk in Communities study participants. PA was assessed using 24-hour/7-day wrist-worn accelerometry and defined using measures of daily activity volume, fragmentation, and variability. Overall and domain-specific cognitive function were derived using mixed-effects models and divided into tertiles of cognitive decline (fast, slow, stable(ref)). Associations between cognitive trajectories and PA were analyzed using multivariable linear regression adjusted for relavant covariates. At baseline, fast cognitive decliners were more likely to have low education, smoking, and ≥1 APOE ε4 allele compared to those in the slow-decline and stable groups. For overall cognition, compared to those in the stable group, those in the fast- and slow-decline groups had lower daily activity (β=-35.4(SE = 9.8)min/day, β=-24.3(SE = 8.6)min/day, respectively), higher fragmentation (β = 2.9%(SE = 0.7), β = 1.3%(SE = 0.6), respectively), and lower variability of activity (β=-127.6(SE = 18.4), β=-62.9(SE = 16.2), respectively) (p < 0.01 for all). In domain-specific analyses, results for executive function were similar to overall cognition, but significance for language and memory results varied. Across all domains, variability was consistently lower among those in the fast-decline group compared to the stable group (p < 0.05 for all). Collectively, these results suggest those with faster cognitive decline have lower and more variable daily PA, independent of baseline cognition. Notably, variability of PA may be an indicator of adverse cognitive change across domains.

